# Derivation and external validation of dendritic cell-related gene signatures for predicting prognosis and immunotherapy efficacy in bladder urothelial carcinoma

**DOI:** 10.3389/fimmu.2022.1080947

**Published:** 2022-12-12

**Authors:** Bingzheng An, Zhaoxin Guo, Junyan Wang, Chen Zhang, Guanghao Zhang, Lei Yan

**Affiliations:** ^1^ Department of Urology, Qilu Hospital of Shandong University, Jinan, China; ^2^ Department of Biostatistics, University of Michigan School of Public Health, Ann Arbor, MI, United States

**Keywords:** bladder urothelial carcinoma, immunotherapy efficacy, somatic mutation analysis, dendritic cells-mediated immune, RYR2 mutation

## Abstract

**Background:**

In the regulation of tumor-related immunity, dendritic cells (DCs) are crucial sentinel cells; they are powerful to present antigens and initiate immune responses. Therefore, we concentrated on investigating the DC-related gene profile, prognosis, and gene mutations in bladder urothelial carcinoma (BLCA) patients to identify sensitivity to immunotherapy of patients.

**Methods:**

According to DC infiltration, BLCA patients were divided into two subgroups, and differentially expressed genes (DEGs) were obtained. Patients were classified by unsupervised clustering into new subgroups. The least absolute shrinkage and selection operator (LASSO) regression analysis and Cox regression were used to develop a DC-related risk model. CIBERSORT, xCell, and GSEA were used to infer immune cells’ relative abundance separately and enriched immune pathways.

**Results:**

A total of 29 prognosis-related DEGs were identified from the unsupervised cluster. Among them, 22 genes were selected for constructing the DC-related risk model. The dendritic cell-related risk score (DCRS) can accurately distinguish patients with different sensitive responses to immunotherapy and overall survival outcomes. Furthermore, patients with ryanodine receptor 2 (RYR2) mutation had a better prognosis.

**Conclusions:**

The DCRS played an essential part in immunity pathway and formation of TME diversity. Our study indicated that RYR2 mutation combined with DCRS is useful for predicting the prognosis and discovering appropriate patients for immunotherapy.

## Introduction

Bladder urothelial carcinoma (BLCA) is one of the most common cancer types of the genitourinary tract, with an increased incidence in younger age (1). The latest statistics reveal that the incidence and mortality rates of BLCA are 80.5/100,000 and 32.9/100,000, respectively (2). Surgical resection is the main therapy for BLCA treatment, but the patients after surgery still have a poor prognosis with recurrence, including biochemical recurrence (BCR) and distant metastases. In recent years, immunotherapy such as PD-1/PD-L1 and CTLA4 immune checkpoint inhibitors (ICIs) has been applied in clinic, which brings hope for cancer patients (3). A sound performance of anti-tumor activity was manifested by the ICIs in the therapy of most solid tumors, such as melanoma, kidney cancer, lung cancer, and liver cancer (4). However, the low effectiveness of immunotherapy is a challenge in the clinical treatment of bladder cancer. A series of biomarkers have been confirmed to predict the effectiveness of ICI treatment, including the expression level of TMB and PD-L1, neoantigens, intestinal flora, and immune cell status. In fact, the immune status of cancer predicts the effect of immunotherapy. Therefore, we constructed an immune signature to predict the response to immunotherapy in BLCA patients.

The mechanisms of immunotherapy mainly included restarting and maintaining the immune cycle and restoring the anti-tumor immune response in the body (5). The tumor microenvironments consist of malignant tumor cells, fibroblasts, glial cells, and especially immune cells that were a key part in influencing response to immunotherapy. For example, dendritic cells (DCs) activate the release of damage-associated molecular patterns (DAMPs) by delivering, processing, and presenting tumor-associated antigens (TAAs) on the DC surface. Induced by DAMP release, chemokines, cytokines, and interferons (IFNs) are driven to stimulate immune response, which play a crucial role in promoting this process of immunogenic cell death (ICD) (6, 7). DCs are one of the most important immune modulatory cells (8) and a sentinel of the immune system with a unique ability to activate T lymphocytes (9). DCs can drive the activation and development of immunity or immune tolerance. However, the specific functions of these DCs in immune tolerance or development of immunity are unclear (10, 11).

Tumor mutation burden (TMB) was recognized as a potential biomarker related to response to immunotherapy (12). In fact, it has been proven that TMB is more significantly correlated with response rate than PD-L1 expression in immunohistochemistry (13). Tumor protein P53 (TP53) and ataxia telangiectasia and Rad3-related (ATR) mutations related to genomic instability can result in an elevated mutation rate in the tumor genome (14, 15). In addition, tumor-specific neoantigens caused by somatic mutations can also be used as a biomarker named tumor neoantigen burden (TNB) to predict the efficiency of immunotherapy (16). However, only a few tumor mutation burden will generate immunogenic neoantigens and only a few mutant peptides on cell surface are capable of eliciting an immune response (17–19). Consequently, this research for new TMB may further help predict the efficacy of immunotherapy.

In this article, the degree of DC infiltration was evaluated. Based on TCGA-BLCA and GEO data containing the expression files and clinical information of BLCA samples, we constructed a dendritic cell-related risk score (DCRS) model through Cox regression and least absolute shrinkage and selection operator (LASSO) regression analysis. This gene signature has the potential to predict immunotherapy susceptibility in BLCA patients. Furthermore, we explored the alterations in TMB among patients with varying DCRS and combined TMB and DCRS to choose suitable patients for ICI treatment more accurately.

## Method

### DC data acquisition

In this study, a database of 404 patients with BLCA RNA-seq (level 3) data with clinical data were obtained from The Cancer Genome Atlas (TCGA; https://cancergenome.nih.gov); additionally, GSE32894 (*n* = 224), which was used to verify the DC-related risk model, was collected from the GEO database (http://www.ncbi.nlm.nih.gov.geo). The mutation data consisted of 404 BLCA patients obtained from TCGA, and the mutation information of two DCRS group subsets was analyzed by the maftools package (20). All data were freely available from the online website.

### Immune cell infiltration

xCell and CIBERSORT were used to calculate the abundance of infiltrating immune cells in TME. CIBERSORT was based on support vector regression and achieved accurate deconvolution of complex cellular mixtures (21). xCell can calculate the degree of infiltration of 64 immune cells using a set of 10,808 genes (22). Compared with the CIBERSORT algorithm, the activation state of DC cells in the tumor immune microenvironment can be further accurately distinguished.

### Development and verification of a DC-related signature

To better evaluate the immune pathway and immune infiltration pattern of BLCA, a DCRS was established using immune function clusters and different immune infiltration. The constructional procedure of DCRS was as follows: According to the median degree of DC infiltration calculated by xCell, BLCA patients were divided into the DC infiltration degree high (DCH) group and the DC infiltration degree low (DCL) group. The limma package was used to identify differentially expressed genes (DEGs) from the DCL group and the DCH group with false discovery rate (FDR)< 0.05 and logFoldChange > 2. Univariate Cox regression was used to analyze the prognosis of DEGs, with the limiting condition of *p*-value< 0.01. The BLCA patients were divided into two new subgroups, Cluster 1 and Cluster 2, using the ConsensusClusterPlus package. DEGs from Cluster 1 and Cluster 2 were identified using the limma package with |logFC| > 1 and FDR< 0.05. Then, univariate Cox regression was used to identify the prognosis-related DEGs, with the limiting condition of *p*-value< 0.01. Afterward, the expression level of each gene and the corresponding coefficient were assembled using LASSO regression analysis to calculate the DCRS, and the formula was as follows:


$$\text{DCRS = }\sum {\text{(Coef}}_{\text{DEGs}}\times {\text{Exp}}_{\text{DEGs}})$$

where Coef_DEGs_ represents the LASSO coefficients and Exp_DEGs_ represents the expression levels. All BLCA samples were divided into DC-related risk score high (DCRS > median value, DCRSH) and DC-related risk score low (DCRS< median value, DCRSL) groups.

### Predicting the response to immunotherapy

The immunophenoscore (IPS) data were collected from The Cancer Immunome Atlas. The quantitative score named IPS can represent tumor immunogenicity and was scored from 0 to 10 points. The response to ICI treatment that was positively correlated with tumor immunogenicity can also be predicted by IPS (23).

### Gene set enrichment analysis

Gene set enrichment analysis (GSEA) was used to identify obvious signaling pathways between the DCRSL group and the DCRSH group; the C5 GO ALL gene set collection was downloaded from the molecular signatures database (MSigDB, v7.5.1). Additionally, gene set variation analysis (GSVA) was used to identify obvious signaling pathways between the BLCA DCH group and the DCL group.

### Statistical analysis

All statistics were accomplished using the R software (version 4.2.1). Time‐dependent receiver operating characteristic (ROC) curve was built using the survival package. LASSO regression analysis was performed using the glmnet package. Univariate Cox regression analysis was calculated using the Survival package. Kaplan–Meier curves of overall survival (OS) were calculated between the two groups using the Survival package and the SurvMiner package. RNA-seq and mutation data were processed by limma and maftools packages. If there are no additional instructions, a *p*-value< 0.05 was considered to be statistically significant for all.

## Results

### Identification of DC-related differentially expressed genes associated with BLCA prognosis

**Figure 1:**
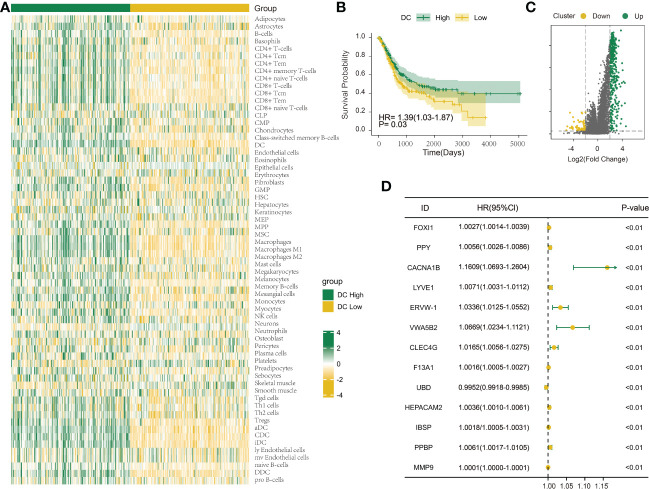
Investigation of the DC infiltration-associated expression change in bladder urothelial carcinoma (BLCA). **(A)** Heatmap showing the degree of infiltration of 64 immune cell types in the TCGA-BLCA cohort (n = 404) using xCell based on a set of 10,808 genes. **(B)** Survival analysis indicated that patients assigned to the two clusters [DC High (n = 202) and DC Low (n = 202)] had significantly different survival outcomes in the TCGA-BLCA cohort. **(C)** Volcano map shows 396 differentially expressed genes between DC High and DC Low. Green dots indicate upregulation and yellow dots indicate downregulation. **(D)** Univariate Cox regression analysis was used to screen genes associated with clinical prognosis with a p-value< 0.01 in the TCGA-BLCA cohort.

**Figure 2:**
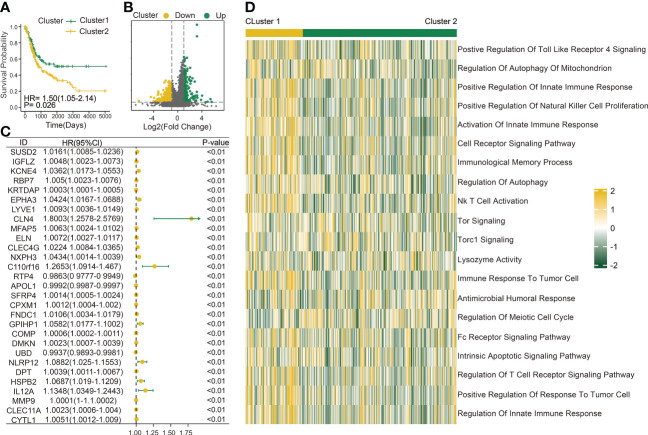
Construction of a DC-related risk model to predict BLCA prognosis. **(A)** Survival analysis of Cluster 1 (n = 110) and Cluster 2 (n = 294). **(B)** Volcano map shows 429 genes differentially expressed between Cluster 1 and Cluster 2. Green dots indicate upregulation and yellow dots indicate downregulation. **(C)** Univariate Cox regression analysis was used to screen genes associated with clinical prognosis with a p-value< 0.01 in the TCGA-BLCA cohort. **(D)** Gene set variation analysis (GSVA) enrichment analysis of the activation states of biological pathways between Cluster 1 (n = 110) and Cluster 2 (n = 294). These biological processes are shown in the heatmap. Yellow represents the activated pathway, and green represents the inhibited pathway.

The immune infiltration score was calculated for each BLCA sample in the TCGA dataset through xCell methodology. Then, the median enrichment score of DC cell infiltration was determined to divide BLCA samples into two groups (DCL and DCH) (**Figure 1A**). Additionally, the ESTIMATE package was used to estimate immune score, estimate score, tumor purity, and stromal score. We found that immune score, estimate score, and stromal score and the extent of infiltrating immune cells were obviously higher in the DCH group than in the DCL group, and tumor purity was obviously lower (**Supplementary Figures S1A–D**). In addition, samples in the DCH group correspond to favorable survival outcome (**Figure 1B**). The result of difference analysis of transcription data showed that 396 DEGs were upregulated (*n* = 339) or downregulated (*n* = 57) in BLCA (**Figure 1C**). Moreover, using univariate Cox regression analysis with *p*< 0.01 as criteria, a total of 13 DEGs were obtained as the prognostic factors (**Figure 1D**). Based on the above results, we speculated that the 13 DEGs are significant factors associated with prognosis and DC. These DEGs were subjected to BLCA patients divided into two new subgroups using the ConsensusClusterPlus package: Cluster 2 (*n * = 294) and Cluster 1 (*n* = 110) (**Supplementary Figures S2A–L**). Survival analysis showed that the prognosis of Cluster 1 was significantly better than that of Cluster 2 (**Figure 2A**). Based on GSVA analysis, we found that DC-associated immune signaling pathways were enriched in Cluster 1, such as the positive regulation of innate immune response, the T-cell receptor signaling pathway, the NK T-cell activation, and the immunological memory process (**Figure 2D**). DCs are essential mediators of the innate immune system (24) and sentinel cells specialized in controlling T-cell function (25), suggesting that the unsupervised cluster contributed to distinct DC-related immune signaling pathways.

### Construction and validation of a DC-related risk model to predict BLCA prognosis

**Figure 3:**
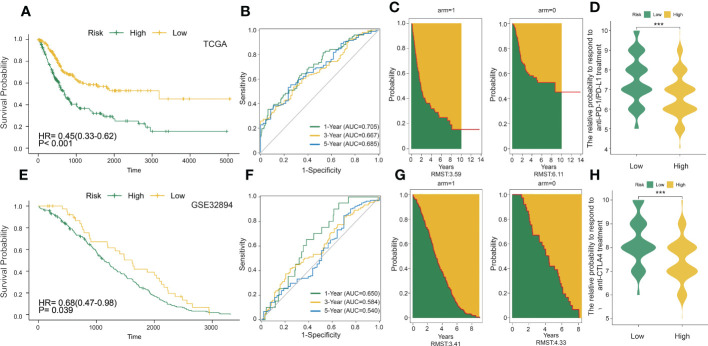
DC-related risk score was constructed in the training set and verified in the validation set. **(A)** Survival analysis of the DCRSL group (n = 202) and the DCRSH group (n = 202) in the training set. In these two groups, the Kaplan–Meier curve with a log-rank p-value< 0.001 showed significant survival differences. **(B)** Time-dependent receiver operating characteristic (ROC) curve analysis of the DCRS in the training set. The 1-, 3-, and 5-year area under curves (AUCs) were 0.705, 0.667, and 0.685, respectively. **(C)** The restricted mean survival (RMS) curve for the DCRS was plotted in the training set. The green part represents the RMS time, and the yellow part represents the restricted mean time lost (RMTL). **(D, H)** The relative probabilities of anti-cytotoxic T lymphocyte-associated protein 4 (CTLA4) and anti-programmed cell death protein 1 (PD-1)/programmed death-ligand 1 (PD-L1) treatment between the DCRSL and DCRSH groups in the training set. The asterisks indicate statistical p-values (***<0.001). **(E)** Survival analysis of the DCRSL (n =43) group and the DCRSH (n = 181) group in the validation set. In these two groups, the Kaplan–Meier curve with a log-rank p-value of 0.039 showed significant survival differences. **(F)** Time-dependent ROC curve analysis of the immune score in the validation set. The 1-, 3-, and 5-year AUCs were 0.650, 0.584, and 0.540, respectively. **(G)** The RMS curve for immune scores was plotted in the validation set. The green part represents the RMS time, and the yellow part represents the restricted mean time lost (RMTL).

To better explore the potential mechanism for the difference of immune status between the two clusters, we compared the expression profiles of two clusters. A total of 429 DC-related DEGs (164 upregulated genes and 265 downregulated genes) were under the conditions of |logFC| > 1 and FDR< 0.05 (**Figure 2B**); subsequently, a list of 29 DC-related DEGs was identified as prognostic factors affecting BLCA prognosis (*p*< 0.01) using univariate Cox regression (**Figure 2C**). A comprehensive and effective DC-related risk model was established to predict prognosis and sensitivity to immunotherapy; the LASSO regression analysis was performed for the 29 DC-related DEGs. When the number of variables (prognostic DEGs) was 22, the likelihood of deviation was minimized (**Supplementary Figure S1E**). The regression coefficients of the 22 variables were calculated by the LASSO model (**Supplementary Figure S1F**). Finally, a 22 DC-related signature was conducted, composed of KCNE4, EPHA3, IL12A, RBP7, UBD, KRTDAP, IGFL2, DMKN, NLRP12, C11orf16, MFAP5, COMP, FNDC1, APOL1, DPT, CLEC4G, SUSD2, RTP4, CBLN4, NXPH3, LRP1B, and MMP9. Results showed that this DCRS was significantly higher in patients with advanced TNM stage (**Supplementary Figure S1G**). We defined the DCRSH and DCRSL according to the median value of DCRS. Samples in DCRSH correspond to favorable overall survival (**Figure 3A**). The ROC curves of the DC-related risk model showed a favorable performance of predicting prognosis. The AUC values of 1 year, 3 years, and 5 years were 0.705, 0.667, and 0.685, respectively, all of which were higher than 0.6 (**Figure 3B**). In addition, we utilized the SURVRM2 package to assess the restricted mean survival time (RMS time, the mean survival time of patients at a specific time *t* or the life expectancy of *t* year) for BLCA patients during the follow-up period (**Figure 3C**). The RMS time was 3.59 years for the DCRSH group and 6.11 years for the DCRSL group, which further illustrated the favorable prognosis of the DCRSL group.

The DC-related risk model was further verified in the validation dataset (GSE32894). A total of 224 BLCA samples in GSE32894 were separated into the DCRSH group (*n* = 181) and the DCRSL group (*n* = 43) by using the same cutoff standard. An analogous outcome was shown in the validation cohort in that the adverse prognosis corresponded to DCRSH group (**Figure 3E**). The AUC values for 1-year, 3-year, and 5-year OS rates were 0.650, 0.584, and 0.540, respectively (**Figure 3F**). The RMS time of the DCRSH and DCRSL groups was 3.41 years and 4.33 years, respectively (**Figure 3G**). The above findings confirmed that the DCRSL group had a better prognosis than the DCRSH group, which supported the idea that the DC-related risk model had the potential to predict the overall survival of BLCA patients.

### Immune scores and response to ICI treatment in BLCA

Due to the absence of data associated with immunotherapy response in the TCGA database, we extracted two important IPS data to replace patient’s response to immunotherapy from the TCIA database (IPS-PD-1/PD-L1/PD-L2_POS and IPS-CTLA4_POS). The relative probability of responding to anti-CTLA4 and anti-PD-1/PD-L1 treatment was much higher in the DCRSL group (**Figures 3D, H**). These consequences demonstrated that patients in DCRSL had a higher potential to benefit from immunotherapy.

### Genomic features, molecular functions, and mechanisms of DCRSH and DCRSL groups

**Figure 4:**
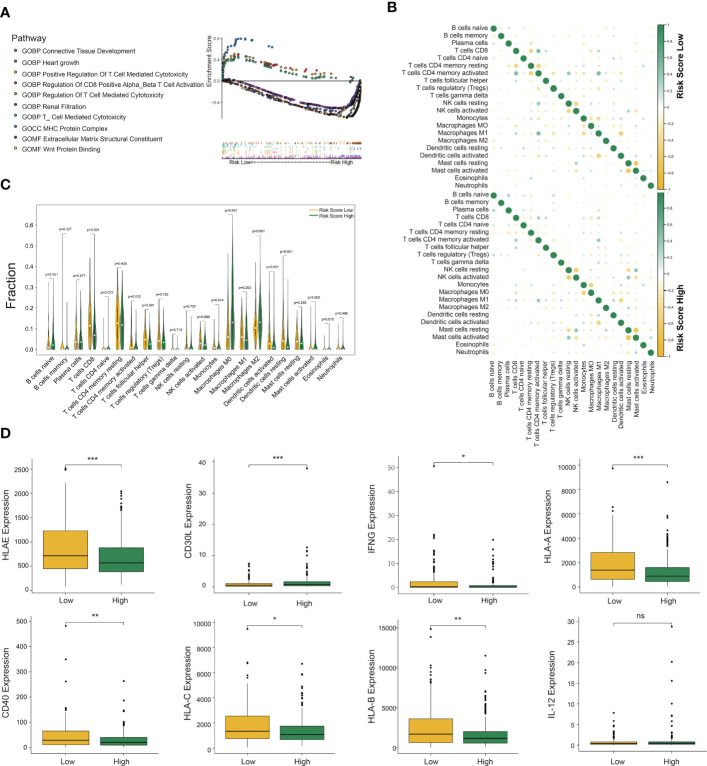
Gene set enrichment analysis (GSEA), immune cell infiltration, and cytokine expression in the DCRSL and DCRSH groups. **(A)** Gene ontology (GO) enrichment analysis of the activation states of immune-related pathways in distinct DC-related risk score groups. **(B)** The relationship between the abundance ratios of different tumor immune-infiltrating cells. **(C)** The horizontal axis and vertical axis represent tumor-infiltrating immune cells and relative percentages, respectively. Yellow and green represent the DCRSL group and the DCRSH group. **(D)** The expression of cytokines associated with DC between the DCRSL group and the DCRSH group. The upper and lower ends of the box indicate the interquartile range of values. The line in the box indicates the median value, and the black dot indicates the outlier. The asterisks indicate statistical p-values (*<0.05, **<0.01, ***<0.001).

As the above findings demonstrated the accuracy of the DC-related risk model, we tried to explore the biological difference between two groups. To reveal the specific biological processes associated with immunity, we performed GSEA GO analysis to calculate the enrichment score of pathways and biological terms in the DCRSH and DCRSL groups (**Figure 4A**). The results showed that the immune pathway in the DCRSL group was associated with the T-cell-mediated cytotoxicity pathway, MHC protein complex, and regulation of CD8^+^ T-cell activation. Therefore, we speculated that DCRS could distinguish the DC-associated immune status and predict the prognosis.

### Immune cell infiltration in different DC-related risk groups

The CIBERSORT algorithm was able to calculate the abundance of infiltrating immune cells and has been widely applied in the preceding studies related to the tumor immune microenvironment. Therefore, we used the CIBERSORT algorithm to assess the infiltration levels of 22 immune cells. The results with *p*-values less than 0.05 are shown in **Supplementary Figure S1H**. In the DCRSL group, the correlation among immune cells was higher (**Figure 4B**). M1 macrophages had a significantly negative correlation with DCs activated in both the DCRSH and DCRSL groups. Furthermore, in the DCRSL group, activated NK cells had the highest positive correlation with activated DCs. The infiltration levels of immune cells between the DCRSH and DCRSL groups are shown in **Figure 4C**. The estimated proportion of naive B cells (*p*< 0.001) and M0 (*p*< 0.001) and M2 (*p*< 0.001) macrophages was significantly higher in the DCRSH group, while CD8^+^ T cells (*p*< 0.001), activated CD4^+^ memory T cells (*p* = 0.012), follicular helper T cells (*p*< 0.001), activated mast cells (*p* = 0.005), resting DCs (*p*< 0.001), activated DC (*p*< 0.001), and monocytes (*p* = 0.014) were significantly enriched in the DCRSL group.

The above results showed that the immune infiltration level in the DCRSL group was significantly higher than that in the DCRSH group. IFN-γ is a cytokine that promotes the differentiation of DCs (26). We observed an elevated expression level of IFN-γ in the DCRSL group. It is well known that DCs consist of two states, resting state and activated state. In the resting state, DCs express low levels of MHC molecules and B7 molecules on the surface, which is not beneficial when presenting antigens to T cells. In contrast, activated DCs highly express MHC-II/I-like molecules and co-stimulatory molecules (such as B7 and ICAM) (27). Although activated DCs have a weak ability to uptake and process antigens, they are powerful to present antigens and initiate immune responses (28). Importantly, activated DCs can regulate cellular differentiation and activation of T cells and especially promote the recruitment and activation of CD8^+^ T cells that were associated with the immunotherapy response. We compared the expression of MHC molecules and cytokines secreted from DCs in two groups. We found high expression of MHC molecules in the DCRSL group. The expression of CD40 and CD30L, which mediate central tolerance to Treg cells, was also increased in the DCRSL group. These findings may contribute to explain the better prognosis of the DCRSL group (**Figure 4D**).

### Comparison of somatic mutations in the DCRSH and DCRSL groups

**Figure 5:**
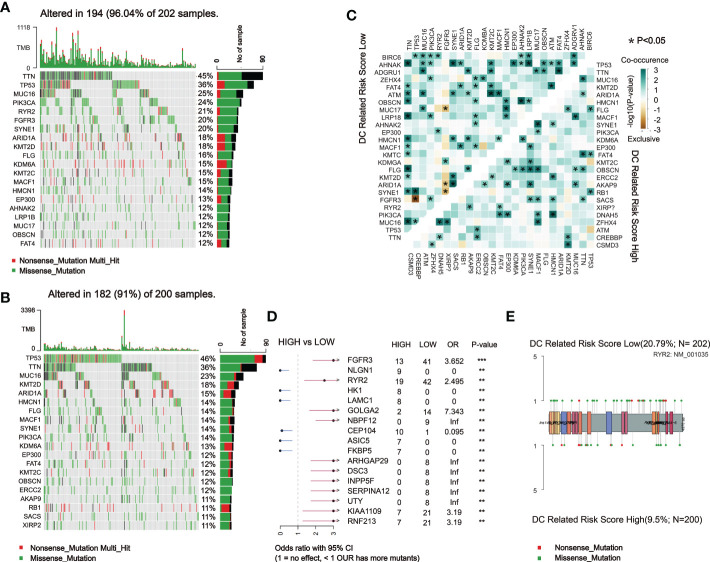
Landscape of somatic mutations between the DCRSL and DCRSH groups. **(A, B)** Waterfall plot of tumor somatic mutations established by those with low DC-related risk scores (n = 202) **(A)** and high DC-related risk scores (n = 200) **(B)**. **(C)** The heatmap shows the mutual co-occurring and exclusive mutations of the top 20 frequently mutated genes. The color or symbol of each cell represents the statistical significance of the exclusivity or co-occurrence of each pair of genes, respectively. Green represents mutual co-occurrence, and brown represents exclusive mutation. Asterisks indicate statistical p-values (*<0.05). **(D)** Forest plot of statistically significant mutant genes between the groups. Asterisks indicate statistical p-values (*<0.05, **<0.01, ***<0.001). **(E)** The lollipop plot illustrates the differential distribution of variants for ryanodine receptor 2 (RYR2).

**Figure 6:**
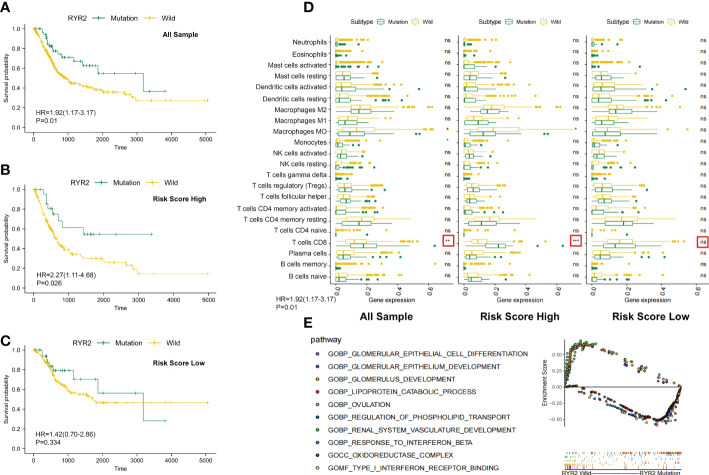
Changes between the RYR2 mutation group and the RYR2 wild group. **(A-C)** Kaplan–Meier curves show the independent relevance between the overall survival time and RYR2 mutation in the DCRSL group (n = 202), DCRSH group (n = 200), and all cohorts (n = 402). **(D)** Effect of RYR2 mutation on tumor immune cell infiltrating in the DCRSL group (n = 202), DCRSH group (n = 200), and whole samples (n = 402) (p-value, *< 0.05, **< 0.01, ns > 0.05). **(E)** Gene set enrichment analysis comparing the RYR2 phenotype between the mutation group and the wild-type group.

Subsequently, we analyzed somatic mutations to further explore genetic differences between the DCRSH and DCRSL groups. Waterfall plots showed the highly mutated genes in the DCRSH (*n* = 200) and DCRSL groups (*n* = 202), where missense mutation was the most common mutation type. Overall, the DCRSH group exhibited a higher number of mutations than the DCRSL group. The top five genes with mutation frequencies were TTN, TP53, MUC16, ARID1A, and KMT2D in the DCRSH group, and in the DCRSL group, the top five genes were TTN, TP53, MUC16, PIK3CA, and RYR2. The most common mutation type was missense mutation in both groups (**Figures 5A, B**). The mutation frequency of the TP53 gene was higher in the DCRSH group, while the mutation frequencies of PIK3CA, RYR2, and TTN genes were lower than those of the DCRSL group. In the two cohorts, SYNE1-TP53 and SYNE1-MACF1 showed significant co-occurrence (**Figure 5C**). This phenomenon suggested that they may have synergistic effects. Additionally, we detected the DEGs with *p*-value< 0.05 using Fisher’s test (**Figure 5D**). Furthermore, we explored the impact of these genes with high mutation frequencies on prognosis in two cohorts. As a result, only the RYR2 mutation had a significant effect on BLCA prognosis in the entire cohort (**Figure 6A**). We further compared the association between RYR2 mutation and prognosis in the DCRSH and DCRSL groups and found that RYR2 significantly affected the prognosis only in the DCRSH group (**Figures 6B, C**).

### Research of the RYR2 mutation pattern in the DCRS cohort

RYR2 encodes Ca^2+^ release channels in sarcoplasmic reticulum, which plays a central role in cardiac excitation–contraction coupling (29). Traditionally, RYR2 mutations were commonly considered to be associated with heart failure and arrhythmias. However, current studies had revealed a correlation of RYR2 mutation with immune cells and tumor progression. The complex communication mechanism between RYR2 and immune cells or immune-related molecules has been identified (30). In addition, it has been proven that RYR2 was associated with malignant progression of triple-negative breast cancer. In the DCRSH group, the mutational frequency of RYR2 was less (10%) than that of the DCRSL group (21%). Additionally, by calculating immune cell infiltration among the DCRSH group, the whole sample group, and the DCRSL group, we found that RYR2 mutation increased CD8^+^ T cells’ infiltration in the whole sample group and the DCRSH group (**Figure 6D**). Coincidentally, survival analysis showed that the prognosis of the RYR2 wild group was significantly better than that of the RYR2 mutation group in the DCRSH group and the whole sample group (**Figures 6A-C**). Furthermore, we used GSEA to search the potential signaling pathway differences between RYR2 mutants and the RYR2 wild group. RYR2 mutations were mainly enriched in type 1 IFN receptor binding and the response to IFN-β. RYR2 wild type was mainly enriched in the glomerulus development and the ovulation pathway (**Figure 6E**).

## Discussion

With the expanded application of immunotherapy, the ICIs are increasingly being studied in cancer treatment. However, only 20%–30% of BLCA patients may benefit from immunotherapy due to the complex regulatory mechanisms among various immune cells in the TME. The current study based on the CD8^+^ T cell-associated immune checkpoint was still far from being adequate to accomplish the desired therapeutic curative effect. In the regulation of tumor-related immunity, DCs are crucial sentinel cells; they are powerful to present antigens and initiate immune responses. DCs are responsible for the delivery, processing, and presentation of TAAs; activation of DAMP release; and promoting the immune stimulative role of chemokines, cytokines, and IFNs. In this study, we aimed to construct a DC-related risk model and identify DC-related genes associated with tumor mutation and immune cell infiltration in TME and accurately identify suitable patients who might benefit from immunotherapy.

In the present study, we used the xCell algorithm to calculate the estimated proportion of immune cells for each BLCA sample. Then, according to the median value of the DC infiltration score, BLCA patients were divided into the DCH and DCL groups. Comparing the DCH and DCL groups, we identified DEGs that were considered as DC-related DEGs. Later on, univariate Cox regression analysis was conducted to select DEGs correlated to prognosis. Based on the expression profiles of prognostic DC-related DEGs, BLCA patients were clustered into two groups (Cluster 1 and Cluster 2) using the ConsensusClusterPlus package. The overall survival of Cluster 2 was obviously reduced compared to that of Cluster 1; GSVA revealed that DC-associated pathways were significantly enriched in Cluster 1 such as the positive regulation of innate immune response and the T-cell receptor signaling pathway. These findings indicated that patients in different unsupervised clusters have different DC-related immune status.

After that, we identified DEGs between Cluster 1 and Cluster 2 groups to better explore the potential mechanism for the difference of immune status between Cluster 1 and Cluster 2, followed by univariate Cox regression analysis to identify prognostic DEGs. We constructed a DC-related risk model using LASSO regression analysis and calculated the DCRS according to the LASSO coefficient. In this research, patients from TCGA were stratified into two groups according to the median value of DCRS and our findings were validated in the GSE32894 cohort. The ROC curves showed that the AUC values of 1 year, 3 years, and 5 years were 0.705, 0.667, and 0.685, respectively, which were higher than 0.6. The higher RMS time in the DCRSL group also proved the validity and the accuracy of DC-related risk model to predict the prognosis of BLCA patients. GSEA also revealed that immune-related pathways were enriched in DCRSL. In conclusion, these discoveries suggested that DCRS may be a promising prognostic indicator of BLCA.

An intimate connection was found between the response to immunotherapy and immune cell infiltration. M2 macrophages had been shown to advance tumor development and were linked to poor overall survival in BLCA patients, and M1 macrophages are capable of antigen presentation and promoting inflammatory responses (31). An obvious rise in degree of infiltrating M0 (*p*< 0.001) and M2 (*p*< 0.001) macrophages had been observed in the DCRSH group, which was found to have a worse prognosis, while in the DCRSL group, the abundances of infiltrating other immune cells were greater, such as CD8^+^ T cells (*p*< 0.001), activated CD4^+^ memory T cells (*p* = 0.012), follicular helper T cells (*p*< 0.001), activated mast cells (*p* = 0.005), resting DCs (*p*< 0.001), activated DCs (*p*< 0.001), and monocytes (*p* = 0.014). Meanwhile, patients in the DCRSL group may have increased sensitivity to immunotherapy. In addition, we also found a high expression of MHC-related molecules in the DCRSL group. Generally, MHC-related molecules’ high expression was associated with the activation of antigen presentation in DC cells and the initiation of immune response; these results may explain the better prognosis of the DCRSL group. Collectively, it may imply that the DCRS model might predict clinical response to immune therapy. Although the DC-related risk model showed an effective performance in predicting prognosis, the degree of accuracy was not much higher, which may result from the insufficient number of samples. The model’s accuracy may be further enhanced by expanding the sample size and required validation in a clinical trial with a large sample size.

The recent research illustrated that tumor mutations are associated with the tolerance or response to immunotherapy; therefore, comprehensive genomic mutation analyses must be considered to accurately select patients who are suitable for immunotherapy. The mutant landscape was compared in the DCRSL and DCRSH groups. We found that the gene mutation frequency was higher in the DCRSL group. TTN and TP53 had higher mutation frequencies in both groups. It was discovered that TP53 played a vital role in bladder cancer development (32). However, except for RYR2, other genes with high mutation frequencies were not associated with BLCA prognosis whether in risk groups or the whole sample group. RYR2 was traditionally considered to be associated with heart failure and arrhythmias, but recently, some studies (28) have identified complex communication mechanisms between RYR2 and immune cells and immune-related molecules. Nevertheless, the immune regulation mechanism of RYR2 was not clear. In the DCRSH group, the mutational frequency of RYR2 was less than that of the DCRSL group. By comparing the infiltration of immune cells among the DCRSH group, the DCRSL group, and the whole sample group, we found an increased infiltration of CD8^+^ T cells in the DCRSH group and the whole sample group. RYR2 mutations were mainly enriched in the type 1 IFN receptor binding pathway and the response to IFN-β pathway by GSEA. IFN, produced primarily by DCs, has pleiotropic impacts on the immune system (33). The mutation frequency of RYR2 was lower in the DCRSH group. These results suggested that RYR2 mutations may participate in the induction and maintenance of anti-tumor immune responses mediated by DCs. Therefore, we believe that combining DCRS and RYR2 mutations could help to screen BLCA patients who were suitable for immunotherapy.

## Conclusion

In conclusion, our results indicated that patients with low DCRS had a better prognosis and predicted benefit from immunotherapy. These DC-related gene signatures may be valuable for the prognostic stratification and patient selection of ICI before treatment. At the same time, it was expected that the DCRS model would improve our understanding of TME and the genomic features and guide immunotherapy and combination therapy strategies.

## Data availability statement

The original contributions presented in the study are included in the article/**Supplementary Material**. Further inquiries can be directed to the corresponding author.

## Author contributions

LY and BA designed the study and analyzed the data. BA and ZG wrote the manuscript and performed the data analysis. JW, CZ, and GZ participated in the picture drawing and processing. All authors contributed to the article and approved the submitted version.

## Supplementary material

Supplementary Figure 1 Immune infiltration and the least absolute shrinkage and selection operator (LASSO) regression analysis. **(A–D)** The tumor purity, estimate scores, immunity scores, and stromal scores between the DCL group and the DCH group; the line in the box indicates the median value, and the black dot indicates the outlier. The asterisks indicate statistical p-values (***< 0.001). **(E, F)** The least absolute shrinkage and selection operator (LASSO) Cox regression for the DC-related key differentially expressed genes. **(G)** Immune cell abundance ratios in the BLCA cohorts. Each column represents a sample, and each column uses a different color and height to indicate the abundance ratio of immune cells in the sample.

Supplementary Figure 2 Unsupervised clustering grouping in BLCA. (A–J) Unsupervised clustering algorithm in BLCA patients and consensus matrices for k = 2–9.
