# Supplementary figures and images for: Derivation and external validation of dendritic cell-related gene signatures for predicting prognosis and immunotherapy efficacy in bladder urothelial carcinoma
Source: Front Immunol. 2022 Dec 12;13:1080947. doi: 10.3389/fimmu.2022.1080947 (PMC9790929; doi:10.3389/fimmu.2022.1080947)

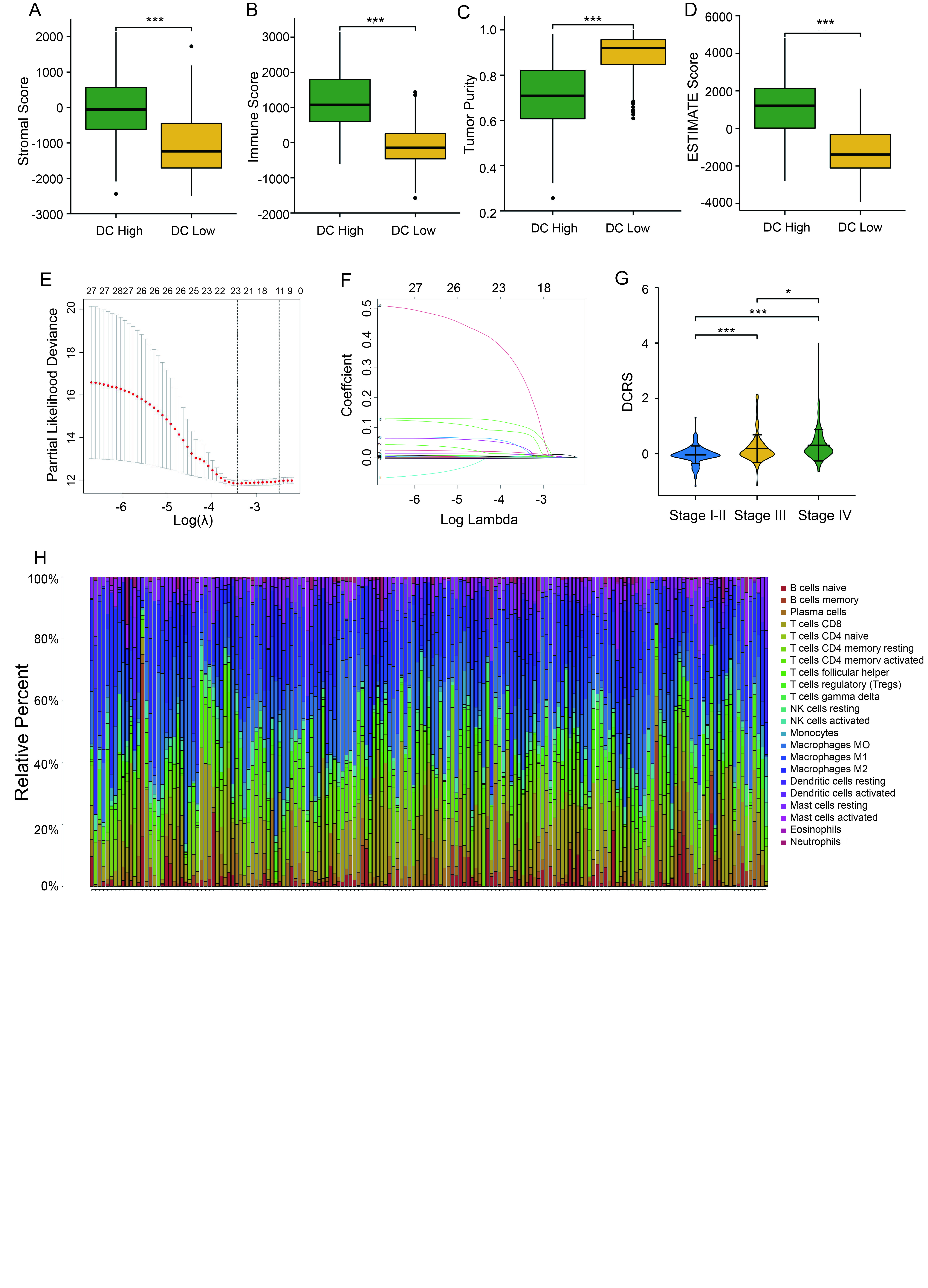

Supplement: Supplementary Figure 1 — Immune infiltration and the least absolute shrinkage and selection operator (LASSO) regression analysis. (A–D) The tumor purity, estimate scores, immunity scores, and stromal scores between the DCL group and the DCH group; the line in the box indicates the median value, and the black dot indicates the outlier. The asterisks indicate statistical p-values (***< 0.001). (E, F) The least absolute shrinkage and selection operator (LASSO) Cox regression for the DC-related key differentially expressed genes. (G) Immune cell abundance ratios in the BLCA cohorts. Each column represents a sample, and each column uses a different color and height to indicate the abundance ratio of immune cells in the sample. [file Image_1.jpeg]

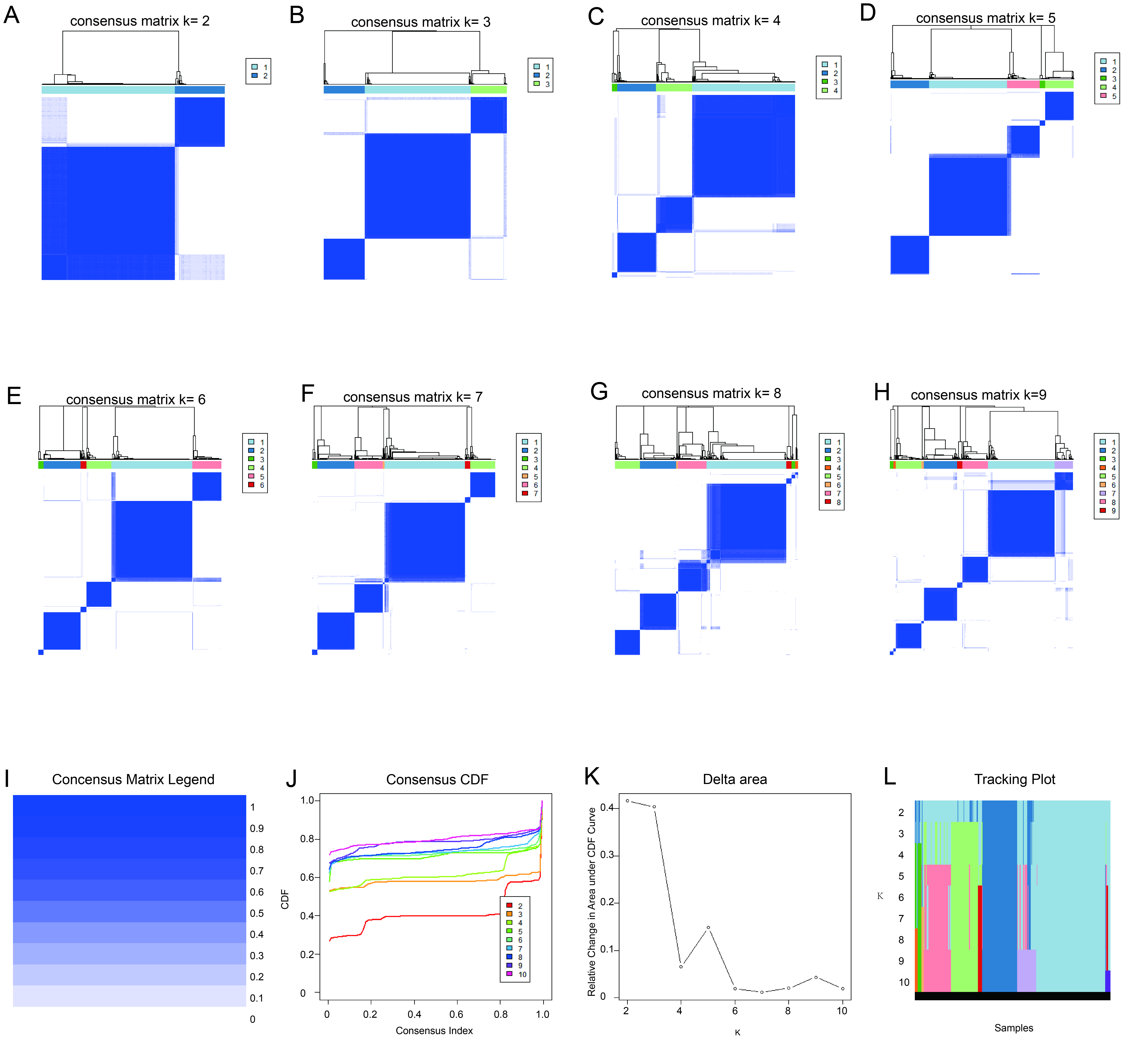

Supplement: Supplementary Figure 2 — Unsupervised clustering grouping in BLCA. (A–J) Unsupervised clustering algorithm in BLCA patients and consensus matrices for k = 2–9. [file Image_2.jpeg]
